# Editorial: Pathogens at the interface of animals in close contact with humans: risks and benefits, with special regard to immunosuppressed people

**DOI:** 10.3389/fvets.2025.1560144

**Published:** 2025-02-05

**Authors:** Martina Jelocnik, José Soares Ferreira Neto, Alda Natale

**Affiliations:** ^1^School of Science, Technology and Engineering, University of the Sunshine Coast, Sippy Downs, QLD, Australia; ^2^Faculty of Veterinary Medicine and Animal Science, University of São Paulo, São Paulo, Brazil; ^3^Experimental Zooprophylactic Institute of the Venezie (IZSVe), Padua, Italy

**Keywords:** zoonoses, veterinary disease, immunosuppressed people, animal pathogen, disease spillover

## Introduction

Zoonotic diseases, caused by a variety of pathogens (such as viruses, bacteria, fungi, and protozoa), continue to be a global concern. The World Health Organization (WHO) estimates that over 60% of all human pathogens are indeed zoonotic, with 75% of new and emerging human diseases caused by zoonotic pathogens. While some zoonotic diseases have demonstrated pandemic potential (such as H5N1 avian influenza, or COVID-19 coronavirus), there is a significant gap in the awareness and perceived risk of more common veterinary zoonotic diseases among both the general population and medical professionals. The goal of this Research Topic was to gather current research on veterinary zoonotic infections and diseases, with a specific focus on assessing the real risk associated with close contact between humans and animals, both in professional and recreational contexts. Immunocompromised people are particularly exposed to zoonoses that are widely spread, but also to minor and emergent pathogens. This Research Topic presents six new papers that expand our understanding of the prevalence of zoonotic pathogens, describe the development of new tools to safeguard against them, and assess the role of veterinary bacterial pathogens in the spread of antimicrobial resistance (AMR) with potential risks to human health.

## Understanding the reservoirs of zoonotic pathogens

Understanding the reservoirs, host range, exposure and variation in shedding rates of zoonotic pathogens in the environment is crucial to deciphering the dynamics of zoonotic spillover at the human-animal-environment interface and informing targeted One Health interventions to reduce disease risk.

Rats are major reservoirs of the pathogenic *Leptospira interrogans* serogroup Icterohaemorrhagiae, the bacteria causing the zoonotic disease leptospirosis. However, the impact of variations in rat abundance and pathogen shedding rates on spillover transmission to humans remains unclear. Soni et al. investigated how spatial variation in animal reservoir abundance and pathogen pressure affects *Leptospira* spillover transmission to humans in an urban informal settlement in Brazil using novel longitudinal eco-epidemiological approaches. Their findings indicate that environmental and hydrological factors play a more significant role in *Leptospira* spillover than individual rat-associated factors.

Similarly, stray cats can potentially act as a reservoir for zoonotic agents, such as *Chlamydia felis*. *Chlamydia felis* is a pathogen with documented zoonotic potential associated with conjunctivitis and/or upper respiratory disease in cats, thereby posing a risk of exposure to both humans and domestic cats. Bellinati et al. sought to identify the molecular occurrence of *Chlamydia* in stray and colony cats in northeastern Italy. A total of 7.7% of cats (29/379) tested positive for *C. felis*, with only one cat exhibiting respiratory symptoms. Although the zoonotic risk of *C. felis* was low across Italy, it would be prudent to exercise caution when handling stray cats, especially for immunocompromised people.

Bovine tuberculosis (bTB), caused by the zoonotic bacteria of the *Mycobacterium tuberculosis* complex, is one of the primary infectious diseases affecting cattle. While several countries have managed to eradicate this zoonotic disease, bTB remains endemic and uncontrolled in many countries in Africa, Asia, Latin America, and the Middle East. Silva Oliveira et al. aimed to identify the risk factors for bTB in the state of Pará, Brazil, to estimate the prevalence of bTB at farm and animal levels, and to verify the existence of heterogeneities between regions through a cross-sectional study.

A total of 976 properties comprising 17,151 animals were surveyed. The prevalence of infected properties in the regions ranged from 3.1% to 18.6%, while tuberculin-positive animals ranged from 0.24% to 4.8%. The implications of these results for both livestock and public health were discussed, and coordinated One Health strategies to control or eradicate the disease were suggested.

## Development of molecular tools for disease surveillance

Mpox (previously known as Monkeypox, MPX) is a zoonotic disease caused by an orthopoxvirus called monkeypox virus (MPXV). MPXV has a wide host range, posing a significant risk of cross-species transmission. The WHO declared the Mpox outbreak a Public Health Emergency of International Concern in 2022. Goatpox virus (GTPV) primarily infects goats and causes contagious skin disease. This infection is characterized by poxviral lesions that strongly resemble the symptoms of MPXV infection. Therefore, accurate, specific and sensitive real-time qPCR assays are the preferred diagnostic method for the rapid detection and differentiation of MPXV and GTPV. Lin et al. described the development of a multiplex real-time PCR assay targeting MPXV and GTPV genes that can simultaneously detect MPXV clades I and II along with GTPV. The assay demonstrated target specificity and high sensitivity, with detection limits at 207.83 copies/reaction for MPXV clade I, 252.07 copies/reaction for MPXV clade II, and 208.72 copies/reaction for GTPV. This assay can serve as a vital tool to safeguard against the MPXV epidemic.

## AMR: a critical global One Health challenge

AMR remains one of the top global public health threats according to the WHO. Wild, domestic and companion animals, particularly dogs and cats, have been identified as potential reservoirs and carriers of AMR bacteria. In their systematic review, Karalliu et al. aimed to assess and compile risk factors associated with the carriage of AMR-*Enterobacterales* in dogs worldwide and to identify relevant knowledge gaps to direct future research. They found that antimicrobial use was the most common risk factor significantly associated with AMR-*Enterobacterales*, followed by raw diet and hospitalization. This review has implications for small animal veterinary practitioners, public health authorities, and researchers who are actively involved in addressing the challenge of AMR.

Livestock farming, in particular pig farms, has been demonstrated to have a role as a significant source and reservoir of AMR bacteria. In addition to meat, soil and wastewater, airborne dust from pig farms may contain AMR bacteria and determinants. Hein et al. aimed to investigate bacterial communities and AMR in airborne dust from pig farms using bacterial isolation, antimicrobial profiling and metagenomic sequencing analyses. Airborne dust, pig feces and feed were collected from nine pig farms in Thailand. The authors showed that *Staphylococcus* and *Enterococcus* species were the most frequently detected bacterial species in all samples, with isolates exhibiting various AMR phenotypes and rates. Metagenomic analysis identified 159 AMR genes from 12 different antibiotic classes. Airborne dust from pig farms contained a diverse range of bacterial species and genes encoding AMR to a range of clinically important antimicrobial agents, indicating a significant role in the spread of AMR bacterial pathogens with potential hazards to human health.

## Summary

This Research Topic demonstrates that the close relationship between humans, animals, and the environment plays a significant role in the transmission and distribution of zoonotic diseases, with zoonotic diseases and/or infections posing serious risks to human health.

